# Social exclusion, integration barriers, and drug-related risk behaviours among immigrants in Europe: a systematic scoping review

**DOI:** 10.3389/fpubh.2026.1834372

**Published:** 2026-06-10

**Authors:** Janice Bemah Sarpong, Anastasia Nanaba Acquah

**Affiliations:** Department of Global Development and Planning, University of Agder, Kristiansand, Norway

**Keywords:** drug-related risk behaviours, Europe, immigrants, integration barriers, scoping review, social exclusion, substance abuse

## Abstract

**Background:**

Europe has witnessed a significant increase in immigration over the last 20 years, which has been associated with complex public health issues. The immigrants, such as refugees, asylum seekers, and economic migrants, are exposed to various social exclusion processes and integration barriers that may increase their vulnerability to drug-related risk behaviours. Although there is increasing awareness of this problem, there is a dearth of research synthesising the interplay of social exclusion, integration barriers and substance use among immigrant groups in European settings.

**Purpose:**

The present systematic scoping review sought to identify and map the forms of social exclusion and integration barriers experienced by immigrants in Europe in relation to substance use service access.

**Methods:**

The systematic scoping review was completed as per the Joanna Briggs Institute (JBI) methodology and was reported following PRISMA-ScR guidelines. The searches were conducted in EBSCOhost databases, MEDLINE, Scopus, and Web of Science to find peer-reviewed qualitative and mixed-method studies published in English between 2015 and 2025. These studies were eligible provided that they had investigated the association between social exclusion or integration barriers and drug-related risk behaviours among immigrant populations residing in Europe.

**Results:**

Five out of 412 citations initially retrieved were included in the study. All the studies employed qualitative or mixed-methods designs and were carried out in Norway, Belgium, Germany and across four metropolitan cities in Europe. The included studies predominantly sampled immigrants already in contact with treatment or healthcare services and/or those experiencing homelessness or precarious housing. The synthesised results indicated that these specific immigrant subgroups experience a variety of interrelated barriers that comprise language challenges, stigma and cultural taboos surrounding substance use, discrimination and racism, lack of awareness of existing services, precarious legal and socioeconomic conditions, and inadequate culturally competent care. These barriers were experienced at an individual, community, and systemic level and seemed to contribute to social exclusion and vulnerability to substance use and harm.

**Conclusion:**

The findings suggest that social exclusion and integration barriers are closely associated with access to and experiences with substance use services among specific subgroups of immigrants in Europe. The results highlight the necessity to have culturally sensitive, low-threshold, and linguistically accessible substance use services. Policymakers, service providers, and researchers need to embrace intersectional and participatory strategies to address the structural determinants that may contribute to health inequities among immigrants.

## 1 Introduction

The influx of immigrants into Europe has grown significantly in the last 20 years due to armed conflict, political instability, economic distress, and the pursuit of improved living standards (1, 2). In the European Union and its neighbouring countries, there are millions of immigrants, among them refugees, people seeking asylum, labour migrants and undocumented people. These populations are extremely diverse and vary significantly in terms of the extent to which they are exposed to risk factors, access to services and integration paths, with different but overlapping vulnerabilities at various stages of arrival and settlement (3). Although most immigrants are resilient and make a positive contribution to the host societies, migration and subsequent settlement can subject one to several social, economic, and psychological stressors, which may negatively influence their health and well-being (4–6). This is especially significant in relation to the establishment of substance-use pathways in that, while immigrants may be bringing specific protective factors with them, these may be slowly dissipated over time as a result of living in marginalising conditions.

Substance use and drug-related risk behaviours have become one of the health issues that have been increasingly of concern to immigrant populations (7). The term ‘substance use’ encompasses patterns of hazardous or harmful use of psychoactive substances, including illicit drugs and the misuse of prescription medications (8). Though immigrants usually show lower rates of substance use than host populations, there is evidence that certain groups of immigrants, especially those subjected to some form of traumatic experience before migration, lengthy asylum processes, social isolation, and marginalisation, may be at elevated risk of developing substance use problems in the long run (4, 9).

The social determinants of health framework and minority stress model are two theoretical frameworks that are particularly relevant to understanding the relationship between migration, social exclusion, and substance use. First, the social determinants of health framework (10, 11) suggests that the conditions in which people are born, grow, live, work, and age are the conditions that shape the health inequities, such as substance use. This framework focuses on the structural factors that affect immigrants’ vulnerability to substance use, including poverty, housing insecurity, tenuous legal status, and limited access to services. Second, the minority stress model (12) emphasizes the chronic stress that comes from experiencing discrimination, stigma and prejudice, which can lead to substance use as a coping mechanism. When applied to immigrant groups, this model sheds light on how the cumulative impacts of racism, social exclusion and cultural marginalisation can lead to harmful patterns of substance use. Both of these frameworks offer a logical analytical framework for understanding associations between social exclusion and integration barriers and drug-related risk behaviours in immigrant populations.

Social exclusion, which is generally defined as the process through which individuals or groups are systematically deprived of access to resources, rights and opportunities required to participate in social life (13), has been cited as a determinant of health disparity among immigrants (1). Structural and interpersonal barriers to integration are common among immigrants in European contexts. Some of the structural barriers are restrictive immigration policies, inaccessibility of healthcare and social services, housing instability, and precarious employment (14). Interpersonal barriers encompass discrimination, racism, stigmatisation, language challenges and cultural misinterpretations in health care facilities (15, 16).

The social exclusion and substance use among immigrants in Europe is a poorly studied field. Although single pieces of research have investigated aspects of this relationship, including barriers to substance use treatment among refugees (17) or recovery capital among ethnic minorities (18), no comprehensive synthesis has been conducted investigating the forms of social exclusion and integration barriers to drug-related risk behaviours among immigrant populations in the context of European origin. The knowledge gap is problematic, as without a clear grasp of the structural and societal forces that may contribute to immigrants’ substance use, interventions and policy responses are likely to be fragmented and inadequately focused (19, 20).

The European Union Drugs Agency (EUDA; formerly the European Monitoring Centre for Drugs and Drug Addiction, EMCDDA) has already recognized the necessity to pay more attention to drug use and associated harms in the context of migrant and ethnic minority populations, with an observation that current services usually do not address the needs of these populations (21). Studies have similarly highlighted significant gaps in knowledge regarding substance use among forced migrants, including the lack of culturally competent interventions and the insufficiency of existing services to address the needs of diverse migrant populations (9, 19). These developments highlight the relevance and timeliness of a scoping review that traces the current state of knowledge on the topic of social exclusion, integration barriers, and drug-related risk behaviours among immigrants in Europe. Consequently, this systematic scoping review aimed to identify and map the forms of social exclusion and integration barriers in relation to substance use service access and experiences among specific subgroups of immigrants in Europe.

## 2 Materials and methods

This scoping review was carried out according to the Joanna Briggs Institute (JBI) approach to scoping reviews (22, 23). The scoping review method was chosen due to its specific appropriateness when it comes to mapping complex and new fields of research, pinpointing the essential concepts, and examining the extent and nature of available evidence (24). The study design is suitable considering the multidimensional nature of the social exclusion, integration barriers and drug-related risk behaviours among immigrants in Europe, an area that has not been studied extensively before. Reporting of the review was based on the Preferred Reporting Items of Systematic Reviews and Meta-Analyses Extension of Scoping Reviews (PRISMA-ScR) (25).

### 2.1 Scoping review protocol

A protocol was developed before conducting the review to guide the study objectives, methodology, inclusion and exclusion criteria, data sources, search strategy, and data extraction procedures. The following protocol was discussed and accepted by the research team for transparency purposes and method consistency. The protocol was not registered in a public registry or repository, which is acknowledged as a methodological limitation. The protocol is available from the corresponding author upon request.

### 2.2 Participants

The population of interest included immigrants in the European countries, such as refugees and asylum seekers, labour migrants, undocumented migrants, and individuals with an ethnic minority background and a migration history. There were no age, gender, country of origin, or length of stay restrictions. Healthcare professional and service provider studies were only included if they provided a direct statement on the experiences, barriers, or needs of immigrant populations as it concerns substance use. In such instances, the professional opinions were viewed as supplementary to the immigrant experience rather than as independent conclusions.

### 2.3 Interventions and comparators

Given that this scoping review was exploratory, no particular interventions or comparators were investigated. The review aimed to chart the range of evidence examining the relationship between social exclusion, integration barriers, and drug-related risk behaviours rather than assessing whether certain interventions were effective or not. The rationale for examining these three domains together is grounded in the social determinants of health framework and the minority stress model, which provide a coherent analytical lens for understanding how structural and social factors may shape substance use patterns among immigrant populations.

### 2.4 Search strategy

A comprehensive search strategy was established and applied to more than one database. The keyword search involved the combination of the terms associated with the three core concepts: (1) social exclusion and integration barriers (e.g., “social exclusion,” “discrimination,” “marginalisation,” “integration barriers,” “stigma,” “racism”); (2) drug-associated risk behaviours and substance use (e.g., “substance use,” “drug use,” “substance abuse,” “drug addiction,” “harmful use”); and (3) immigration and migration (e.g., “immigrant,” “refugee,” “asylum seeker,” “migrant,” “ethnic minority”). Search terms were combined using Boolean operators (AND, OR). The full Boolean search strings for each database are provided in Appendix A. The search was restricted to English-language articles published between 2015 and 2025. The searches were conducted between September and December 2024.

### 2.5 Data sources

The search for the information was conducted in the following databases: EBSCOhost (Academic Search Ultimate, CINAHL with Full Text, as well as Psychology and Behavioural Sciences Collection), MEDLINE (through PubMed), Scopus, and Web of Science. Additional screening of the reference lists of the included studies was also done manually to determine additional relevant articles. This hand-searching did not yield any additional studies meeting the inclusion criteria.

### 2.6 Data extraction and study selection

Table 1 summarises the inclusion and exclusion criteria applied in this review.

**Table 1:** Inclusion and exclusion criteria.

Inclusion criteria	Exclusion criteria
(i) Written in English; (ii) Peer-reviewed original research (qualitative or mixed-methods designs); (iii) Study population includes immigrants, refugees, asylum seekers, or ethnic minorities with migration history in European contexts; (iv) Explores forms of social exclusion or integration barriers associated with substance use or drug-related risk behaviours; (v) Published between 2015 and 2025	Studies focused exclusively on non-European settings; Studies restricted to alcohol consumption without reference to other drug-related behaviours; Grey literature (reports, conference abstracts, editorials); Review articles; Quantitative-only study designs

All the retrieved citations were organized and entered into a reference management system, whereby duplicates were eliminated. Titles and abstracts were screened by a single reviewer against the inclusion criteria. To prevent potential selection bias due to single reviewer screening, a second reviewer independently confirmed all inclusion and exclusion criteria at the full-text phase. Disagreements were resolved through discussion and consensus.

Alcohol is included in the umbrella term of substance use, but this review focuses on drug-related risk behaviours that involve illicit drugs and non-prescribed psychoactive substances. Studies that investigated alcohol and other drugs were included, whereas only those that investigated only alcohol were excluded.

A standardised form was used to extract the data. Information extracted was author(s), year of publication, study title, aim/objective, study design and methods, country/context, characteristics of the participants, key findings regarding social exclusion and barriers to integration, key findings regarding drug-related risk behaviours and implications of the findings to practice or policy.

### 2.7 Data analysis

Since the studies included were qualitative, the narrative synthesis approach was used to analyse and present the results (26). The narrative synthesis entailed the identification of common themes and patterns existing in the included studies and areas of divergence. Results were organized thematically based on the major forms of social exclusion and integration barriers recognised and associated with drug-related risk behaviours.

## 3 Results

### 3.1 Flow diagram of study selection

**Figure 1:**
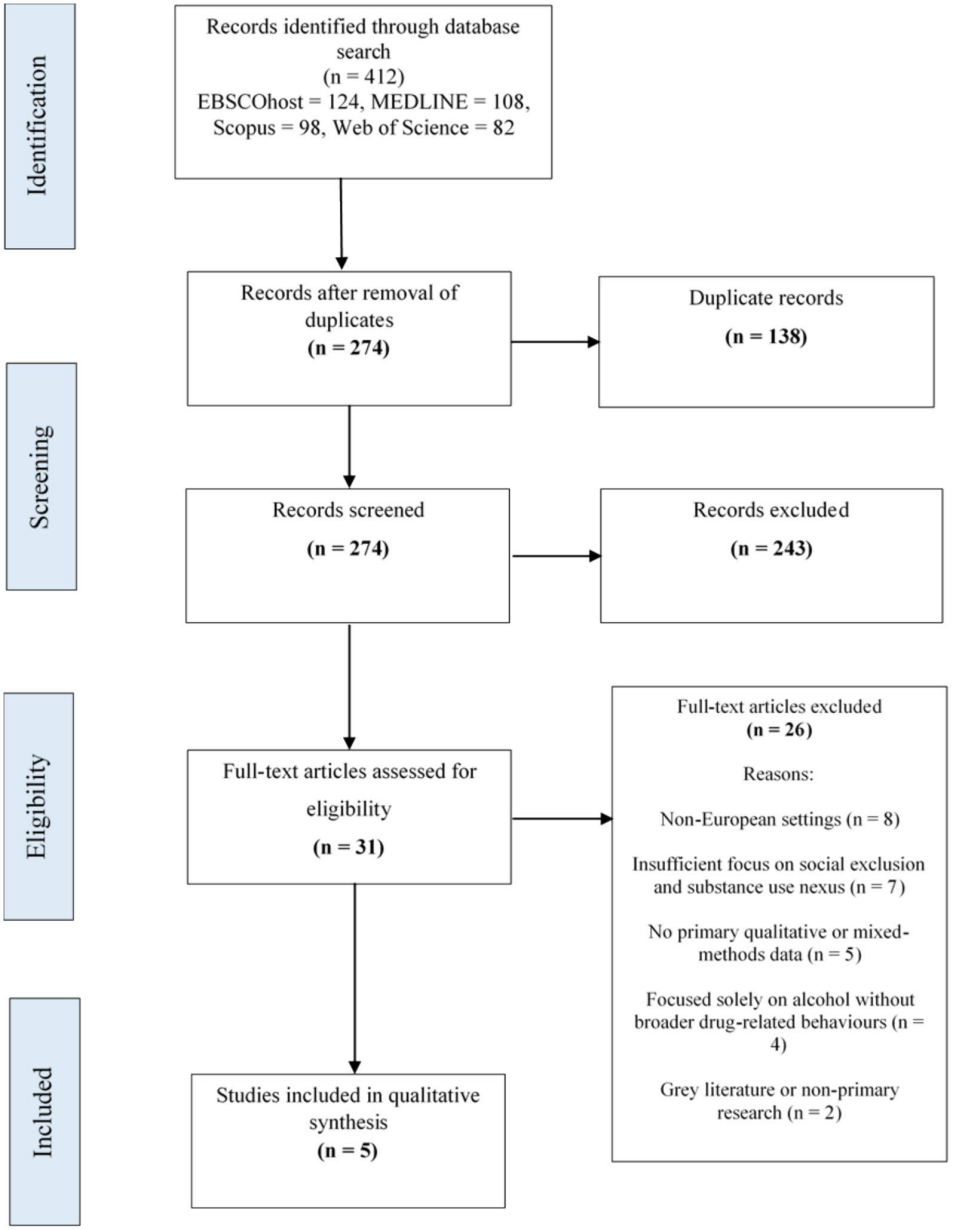
PRISMA-ScR flow diagram of study selection.

The initial search of the EBSCOhost databases, MEDLINE, Scopus and Web of Science produced 412 citations. After removing 138 duplicates, 274 titles and abstracts were screened for eligibility. A total of 243 articles were excluded as they failed to satisfy the inclusion criteria, mostly due to the fact that they failed to focus on the intersection of social exclusion and drug-related risk behaviours among immigrants in Europe. The rest of the 31 full-text articles were assessed based on the eligibility criteria and 26 of them were excluded. Reasons for exclusion were non-European settings (*n* = 8); insufficient focus on the nexus of social exclusion and substance use (*n* = 7); no qualitative or mixed-methods data (*n* = 5); focused solely on alcohol without reference to other drug-related behaviours (*n* = 4); and grey literature or non-primary research (*n* = 2). Five studies were identified that met all the requirements to be included in the scoping review (Figure 1). Hand-searching the reference lists of included studies did not identify any additional eligible articles.

### 3.2 Study selection and characteristics

The five included papers were published between 2017 and 2024 and were carried out in four European countries, including Norway, Belgium, Germany and one across various European metropolitan regions (Amsterdam, Athens, Berlin and Paris). Three studies employed qualitative designs, while two incorporated mixed-methods elements; all studies included qualitative data collection and analysis. The number of participants was between 6 and 99. The participants were immigrants of different regions of origin, such as East Africa, the Middle East, Turkey, Eastern Europe, sub-Saharan Africa, North Africa, and Latin America. Importantly, participants in the included studies were predominantly immigrants already in contact with treatment, recovery, or healthcare services and/or individuals experiencing homelessness or precarious housing situations.

In the article by Pettersen and Debesay (27), the authors employed a qualitative research design based on the interpretive phenomenological analysis (IPA) to conduct the study in Norway on six East African migrants (Somalia, Eritrea, and Sudan) with a history of substance use issues but who had sought assistance within the Norwegian health care system. Pouille et al. (18) examined recovery capital among 34 migrants and ethnic minorities who had recovered from problem substance use in two ethnically diverse cities of Belgium through semi-structured interviews and qualitative content analysis. Pouille et al. (28) used a community-based participatory research (CBPR) design, which involved interviewing 99 drug users with a migration background residing in Amsterdam, Athens, Berlin, and Paris from 43 countries of origin. Saleh et al. (17) explored the barriers to substance use treatment as held by 13 Arabic-speaking refugees and 13 professionals in Germany through in-depth and semi-structured interviews, which were analysed through thematic analysis. De Kock and Decorte (29) focused on problem substance use, discrimination, ethnic identity, and social networks of the individuals with Turkish and Eastern European migration backgrounds in Ghent, Belgium. A summary of study characteristics is given in Table 2.

**Table 2:** Summary of included studies.

Study	Title	Aim/objective	Method and data collection	Sample size	Participant type and status	Context	Substance focus	Key findings
Pettersen and Debesay (27)	Substance use and help-seeking barriers among East African migrants in Norway	Explore help-seeking experiences for substance use	Qualitative; IPA; in-depth interviews	*n* = 6	East African migrants; in treatment/recovery	Norway	Alcohol and illicit drugs	Lack of knowledge, scepticism toward ‘white system’, fear of community exclusion, racism
Pouille et al. (18)	Recovery capital among migrants and ethnic minorities in recovery	Explore recovery capital through lived experiences	Qualitative; semi-structured interviews; content analysis	*n* = 34	Migrants/ethnic minorities in recovery from problem substance use	Belgium (Ghent, Antwerp)	Alcohol and drugs broadly	Stigma, discrimination, poverty, weak networks; cultural/religious recovery assets
Pouille et al. (28)	Challenges and support needs among PMWUD in four European cities	Explore challenges and support needs of PMWUD	Qualitative CBPR; semi-structured interviews	*n* = 99	Persons with migration background who use drugs; many homeless/undocumented	Amsterdam, Athens, Berlin, Paris	Illicit drug use	Childhood adversity, homelessness, lack of documents, loneliness, stigma, linguistic barriers
Saleh et al. (17)	Challenges in substance use treatment: Arabic-speaking refugees in Germany	Investigate SUD treatment challenges	Qualitative; semi-structured interviews & open-ended survey	*n* = 26 (13 refugees, 13 professionals)	Arabic-speaking refugees and service professionals	Germany (Berlin)	Prescription drug misuse and illicit drugs	Language barriers, cultural misunderstandings, stigma/shame, lack of culturally competent care
De Kock and Decorte (29)	Exploring problem use, discrimination, ethnic identity and social networks	Examine substance use, discrimination, identity and networks	Qualitative interviews	Not reported	Turkish and Eastern European migrants with problem substance use	Belgium (Ghent)	Illicit drug use	Weak ethnic identity + discrimination = higher risk; social network quality matters

Source: author’s compilation.

### 3.3 Synthesized findings

The narrative synthesis of the 5 studies that made it through the inclusion criteria demonstrated that there are multiple intertwined themes to the connection between social exclusion, integration barriers, and access to and experiences with substance use services among the sampled immigrant subgroups in Europe.

#### 3.3.1 Language barriers and low health literacy

Language barriers emerged as a frequently reported obstacle to accessing substance use services, identified in three of the five included studies. According to Pettersen and Debesay (27), East African migrants in Norway reported a widespread ignorance concerning the accessible healthcare services and how to make their way through the Norwegian health system. The participants explained that they did not have access to information regarding substance use treatment in their own languages, and thus, they had no idea of the support they had to seek. Saleh et al. (17) reported that the Arabic-speaking refugees in Germany had a hard time navigating the German healthcare system because language barriers not only made communication with the service providers difficult but also led to the incidences of isolation and helplessness. Pouille et al. (28) found the language differences to be a major setback in all four cities considered in Europe, stating that the lack of the ability to communicate effectively with service providers augmented other forms of social exclusion. These results are consistent with the rest of the literature that suggests language barriers to be one of the most influential factors of healthcare access disparities among immigrant communities in Europe (30, 31).

#### 3.3.2 Stigma, cultural taboos, and fear of community exclusion

The stigma of substance use, both amongst the immigrant group and between the immigrant population and the host population, was identified as a theme in four of the five included studies. According to Pettersen and Debesay (27), one of the factors contributing significantly to failure by the East African migrant to seek help in Norway was fear of not fitting in the family or ethnic community. Participants talked about how the use of substances, especially by women, was extremely stigmatised in their communities, and any attempt to seek help was regarded as a disgrace to the family. Pouille et al. (18) also observed that a substance use stigma was also complicated by ethnic minority stigma, which the authors termed accumulated stigma due to overlapping identities, such as ethnicity, substance use, psychological issues, criminal records, poverty, and unemployment. Saleh et al. (17) found that Arabic-speaking refugees in Germany found the use of substances to be very shameful in their cultural background, and this shame extended to seeking professional help. De Kock and Decorte (29) discovered that the perceived discrimination by the host society, together with the low ethnic identity, appeared to increase the likelihood of adhering to further problem substance use, suggesting that stigma may operate not only as a barrier to treatment but also as a factor associated with continued substance use.

#### 3.3.3 Discrimination, racism, and mistrust of health services

Several studies reported experiences of discrimination and racism in healthcare and society as a perceived hindrance to help-seeking and a factor that may contribute to substance use. According to Pettersen and Debesay (27), racism was a unique obstacle and participants identified scepticism in relation to what they termed as a “white system” that they considered not designed to meet their needs. Participants shared the stories of how healthcare professionals treated them differently or disrespectfully, and this undermined their confidence in the health system. Pouille et al. (28) also reported that discrimination and stigma were widely felt in Amsterdam, Athens, Berlin, and Paris, extending beyond healthcare to housing, employment, and interactions with the criminal justice system, creating a cumulative burden of exclusion that participants described as reinforcing substance use as a coping response. De Kock and Decorte (29) found that the perception of discrimination was associated with problem substance use among Turkish and Eastern European migrants in Belgium, especially when individuals did not have a strong sense of ethnic identity and positive social networks. The results are consistent with the existing literature on the relationship between racism and discrimination and poor health among minorities (32).

#### 3.3.4 Precarious legal status, homelessness and socioeconomic vulnerability

The structural conditions of immigrants’ lives, especially precarious legal status, homelessness, and socioeconomic deprivation, emerged as critical factors shaping both substance use patterns and access to care, particularly among the specific subgroups sampled in the included studies. Pouille et al. (28) discovered that in the 99 individuals in the four cities in Europe, a lack of required identity documents was a widespread obstacle in healthcare, social security, and work. Many participants were homeless or in unstable housing, and their drug consumption was inextricably bound to material lack. Pouille et al. (18) emphasized that poverty, lack of access to housing and jobs posed significant barriers to the recovery of migrants and ethnic minorities in Belgium. Saleh et al. (17) also observed that Arabic-speaking refugees in Germany had complex contextual barriers such as unstable accommodation, joblessness, and uncertainty over their legal status, which may have increased their vulnerability to substance use and hindered their engagement with treatment services. These results highlight the importance of social determinants of health in shaping substance use trajectories in immigrant communities (10, 33).

#### 3.3.5 Lack of culturally competent and accessible services

All five studies found that a lack of cultural competence and accessibility of substance use services is a systemic barrier. According to a study conducted by Pettersen and Debesay (27), the East African migrants in Norway found the services that were in existence culturally inappropriate and did not address their unique needs. According to a study by Saleh et al. (17), the professionals in Germany also recognized the absence of culturally sensitive treatment methods and demanded interpreter services, cultural mediators, and community-based prevention methods. Pouille et al. (28) emphasised the importance of low-threshold, outreach-based services with low administrative demands as a way to reach this population. Pouille et al. (18) pointed to the necessity of recovery-supportive environments that optimize the possibility of developing culturally sensitive recovery capital, such as mobilization of cultural and religious resources. De Kock and Decorte (29) proposed services sensitive to overlapping roles of ethnic identity, discrimination, and social network roles in predicting substance use patterns.

### 3.4 Methodological considerations

In line with the scoping review approach (22), no formal critical appraisal of the studies included in the review was carried out using a recognised quality assessment tool. This is suitable for scoping reviews where the purpose is to explore the extent of the evidence and not to evaluate the quality of the studies (24). However, it is acknowledged that all five of the studies included used peer-reviewed journals and had been conducted using a proven qualitative research design. The small number of included studies and the qualitative nature of the evidence limit the generalisability of findings. Self-report data may have been prone to social desirability bias, especially in the context of stigmatised substance use in the populations studied. Additionally, the predominance of participants already in contact with services or experiencing homelessness means that the perspectives of immigrants not engaged with treatment systems are not captured in the available evidence.

## 4 Discussion

This scoping review synthesized five qualitative studies that investigated social exclusion and integration barriers in relation to substance use service access and experiences among specific subgroups of immigrants in Europe. The results indicate the presence of a complicated system of interdependent barriers at the individual, community, and systemic levels.

At the individual level, language barriers and limited health literacy emerged as one of the key barriers to substance use services. Their inability to communicate well with service providers, coupled with the unavailability of information in languages of interest, meant that many immigrants were left without knowledge or access to help (27, 28, 17). These results are consistent with the broader body of research on the disparities in access to healthcare (34, 35).

At the community level, stigma and cultural taboos of substance use generated strong deterrents to help-seeking. The threat of being socially marginalized from their ethnic and religious group, along with the gendered expectations, did not allow many immigrants to acknowledge their issues and seek help (27, 18, 17). This internalised stigma, coupled with perceived host society discrimination, resulted in a two-fold burden that appeared to reinforce isolation and was associated with continued substance use (29). These results are consistent with the stigma theory by Earnshaw (36) that points out that the combination of stigma related to the multiple interacting identities may compound health disadvantages.

At the systemic level, deficiency in culturally competent services, restrictive administrative prerequisites, and broader structural conditions of poverty, homelessness, and precarious legal standing established an environment in which substance use was both a consequence and a reaction to social exclusion (28). The attempts at reducing substance use among immigrant populations will not be adequate without addressing these structural determinants. This inference is echoed by the social determinants of health framework (10, 11). A noteworthy finding was that harm reduction services appeared to play an important role in providing accessible support. Pouille et al. (28) reported the role of the low-threshold harm reduction services as a key point of support entry, addressing basic needs and establishing trust. This suggests that harm reduction strategies, especially those that are delivered through outreach and with cultural sensitivity, may represent **a** promising approach for reaching immigrants with various barriers (21, 20).

Another notable gap that was identified in the review concerns gender-specific experiences. While Pettersen and Debesay (27) emphasized increased stigma among women and Pouille et al. (28) found that the majority of participants were males, none of the studies presented in-depth gendered analysis. This is a notable limitation considering that gender intersects with migration status and ethnicity to influence substance use patterns in distinctive ways (37).

## 5 Limitations

The current scoping review has a number of limitations. The small number of included studies (*n* = 5) indicates that there is little evidence at this intersection, which confirms a significant gap in research while also limiting the synthesis. Screening at the title and abstract stage was conducted by a single reviewer, which may have introduced selection bias, although a second reviewer independently verified all inclusion and exclusion decisions at the full-text stage. The use of English-language publications only could exclude the pertinent studies in other European languages. Although the protocol was developed before the review, it was not publicly registered or deposited in a repository, which limits the ability to assess deviations from the original plan. The immigrant populations represented in the included studies are heterogeneous in some respects (countries of origin, host countries) but notably homogeneous in others: most participants were already in contact with treatment or recovery services and/or experiencing homelessness or precarious housing. This selectivity is a more consequential limitation than heterogeneity of origin alone, as it substantially constrains the transferability of findings to immigrant populations who are not in service contact or not in precarious housing situations. All involved studies are qualitative and rich in insight, but limit generalisability. It should also be noted that two of the five studies included were conducted with participants who used both alcohol and illegal drugs. While these studies were included because they were not restricted to alcohol alone, the synthesised findings do not consistently distinguish between barriers related specifically to illicit drug use and those related to substance use more broadly. This may limit the precision of the review’s conclusions regarding drug-related risk behaviours specifically, and should be considered when interpreting the findings. Lastly, being a scoping review, the study lacked a formal critical appraisal of included studies, as it is common practice (22), which may be considered a limitation.

## 6 Conclusion

The findings of this scoping review suggest that social exclusion and integration barriers are closely associated with access to and experiences with substance use services among specific subgroups of immigrants in Europe, particularly those already in contact with treatment systems or experiencing precarious living conditions. The results indicate that a multi-level strategy should be implemented to address the structural and social determinants of health that may predispose individuals to vulnerability beyond the individual level of intervention. Substance use services should be linguistically accessible, culturally sensitive and designed through the input of the immigrant communities. Low-threshold and outreach-based harm reduction services may represent a promising model for reaching immigrants facing the greatest barriers to care.

The policymakers should focus on eliminating administrative barriers to accessing healthcare, investing in interpreter services and cultural mediation, and educating healthcare professionals on cultural competence. Future studies should explore the intersecting effects of migration, social exclusion, gender, and substance use with specific references to the narratives of immigrants themselves. Participatory research methods should be utilized so that the needs and perspectives become central to service design and delivery.

Lastly, this review highlights the urgency of developing the evidence base. The fact that there are only a few studies that met the inclusion criteria indicates a vital gap that needs to be filled by future research using large-scale studies involving multiple countries using both qualitative and quantitative designs.

## Appendix A

### Full search strategy

The following Boolean search strings were applied across the four databases. All searches were conducted between September and December 2024, restricted to English-language publications from 2015 to 2025.

### EBSCOhost (academic search ultimate, CINAHL, psychology and behavioural sciences collection)

*(“social exclusion” OR “discrimination” OR “marginalisation” OR “marginalization” OR “integration barriers” OR “stigma” OR “racism” OR “xenophobia” OR “structural exclusion” OR “social marginalisation”) AND (“substance use” OR “drug use” OR “substance abuse” OR “drug abuse” OR “drug addiction” OR “substance misuse” OR “harmful use” OR “illicit drug*” OR “drug-related” OR* “substance use disorder”*) AND (“immigrant*” OR “refugee*” OR “asylum seeker*” OR “migrant*” OR “ethnic minorit*” OR “foreign-born” OR “displaced person*” OR “undocumented migrant*” OR “migration background”)*.

### MEDLINE (via PubMed)

*[(“social exclusion”[tiab] OR “discrimination”[tiab] OR “marginali*”[tiab] OR “integration barriers”[tiab] OR “stigma”[tiab] OR “racism”[tiab] OR “xenophobia”[tiab] OR “structural exclusion”[tiab]) AND (“substance use”[tiab] OR “drug use”[tiab] OR “substance abuse”[tiab] OR “drug abuse”[tiab] OR “drug addiction”[tiab] OR “substance misuse”[tiab] OR “harmful use”[tiab] OR “illicit drug”[tiab] OR* “substance use disorder”*[tiab]) AND (“immigrant*”[tiab] OR “refugee*”[tiab] OR “asylum seeker*”[tiab] OR “migrant*”[tiab] OR “ethnic minorit*”[tiab] OR “foreign-born”[tiab] OR “undocumented”[tiab] OR “migration background”[tiab])]*.

### Scopus

*TITLE-ABS-KEY[(“social exclusion” OR “discrimination” OR “marginalisation” OR “marginalization” OR “integration barriers” OR “stigma” OR “racism” OR “xenophobia”) AND (“substance use” OR “drug use” OR “substance abuse” OR “drug abuse” OR “drug addiction” OR “substance misuse” OR “illicit drug*” OR “drug-related” OR* “substance use disorder”*) AND (“immigrant*” OR “refugee*” OR “asylum seeker*” OR “migrant*” OR “ethnic minorit*” OR “foreign-born” OR “undocumented” OR “migration background”)].*

### Web of science

*TS = [(“social exclusion” OR “discrimination” OR “marginalisation” OR “marginalization” OR “integration barriers” OR “stigma” OR “racism” OR “xenophobia”) AND (“substance use” OR “drug use” OR “substance abuse” OR “drug abuse” OR “drug addiction” OR “substance misuse” OR “illicit drug*” OR “drug-related” OR* “substance use disorder”*) AND (“immigrant*” OR “refugee*” OR “asylum seeker*” OR “migrant*” OR “ethnic minorit*” OR “foreign-born” OR “undocumented” OR “migration background”)].*
